# Publisher Correction: An implantable piezoelectric ultrasound stimulator (ImPULS) for deep brain activation

**DOI:** 10.1038/s41467-024-50181-8

**Published:** 2024-07-25

**Authors:** Jason F. Hou, Md Osman Goni Nayeem, Kian A. Caplan, Evan A. Ruesch, Albit Caban-Murillo, Ernesto Criado-Hidalgo, Sarah B. Ornellas, Brandon Williams, Ayeilla A. Pearce, Huseyin E. Dagdeviren, Michelle Surets, John A. White, Mikhail G. Shapiro, Fan Wang, Steve Ramirez, Canan Dagdeviren

**Affiliations:** 1 Media Lab, Massachusetts Institute of Technology, Cambridge, MA 02139 USA; 2 Department of Brain and Cognitive Sciences, McGovern Institute for Brain Research, Massachusetts Institute of Technology, Cambridge, MA 02139 USA; 3 Department of Psychological and Brain Sciences, The Center for Systems Neuroscience, Boston University, Boston, 02215 MA USA; 4 Division of Chemistry and Chemical Engineering, California Institute of Technology, Pasadena, CA 91125 USA; 5 Center for Systems Neuroscience, Neurophotonics Center, Department of Biomedical Engineering, Boston University, 610 Commonwealth Ave., Boston, MA 02215 USA; 6 Department of Neurosurgery, Faculty of Medicine, Istanbul University, Istanbul, 34093 Turkey

**Keywords:** Biomedical engineering, Actuators, Neurological disorders, Electrical and electronic engineering

Correction to: *Nature Communications* 10.1038/s41467-024-48748-6, published online 04 June 2024

The original HTML version of this Article was updated shortly after publication because the previous HTML version linked to an incorrect “Description of Additional Supplementary Files”.

### Supplementary Information

Updated Description of Additional Supplementary Files

