# Supplementary material for: An implantable piezoelectric ultrasound stimulator (ImPULS) for deep brain activation
Source: Nat Commun. 2024 Jun 4;15:4601. doi: 10.1038/s41467-024-48748-6 (PMC11150473; doi:10.1038/s41467-024-48748-6)
Supplement: Supplementary file 1 — Supplementary Information [file 41467_2024_48748_MOESM1_ESM.pdf]

Supplementary Information for

An implantable piezoelectric ultrasound stimulator (ImPULS) for deep brain activation

Jason F. Hou[1]†, Md Osman Goni Nayeem[1]†, Kian A. Caplan[2], Evan A. Ruesch[3], Albit Caban-Murillo[3], Ernesto Criado-Hidalgo[4], Sarah B. Ornellas[1], Brandon Williams[5], Ayeilla A. Pearce[2], Huseyin E. Dagdeviren[6], Michelle Surets[3], John A. White[5], Mikhail G. Shapiro[4], Fan Wang[2], Steve Ramirez[3], Canan Dagdeviren[1]*

Affiliations:

[1]Media Lab, Massachusetts Institute of Technology, Cambridge, MA 02139, United States.

[2]Department of Brain and Cognitive Sciences, McGovern Institute for Brain Research, Massachusetts Institute of Technology, Cambridge, MA 02139, USA.

[3]Department of Psychological and Brain Sciences, The Center for Systems Neuroscience, Boston University, Boston, 02215, MA, USA.

[4]Division of Chemistry and Chemical Engineering, California Institute of Technology, Pasadena, CA, 91125, USA

[5]Center for Systems Neuroscience, Neurophotonics Center, Department of Biomedical Engineering, Boston University, 610 Commonwealth Ave., Boston, MA 02215, USA

[6]Department of Neurosurgery, Faculty of Medicine, Istanbul University, Istanbul, 34093, Turkey.

†These authors contributed equally: Jason F. Hou, Md Osman Goni Nayeem

*Corresponding author. Email: canand@media.mit.edu

This supplementary information contains:

Supplementary Note 1-5, Supplementary Figures 1-23, Supplementary Video 1, and Supplementary References 1-32.

Supplementary Note 1: Rationale for choosing KNN as piezoelectric material for ImPULS

We chose KNN over PZT as the piezoelectric material for ImpULS fabrication. This is because i) KNN is biocompatible ii) has piezoelectric properties comparable or greater than that of PZT (*SI ref 1-3*). A comparison of the d_{33} value and Curie temperature of different piezoelectric materials (including PZT) in the thin film and bulk form is given below:

**Material**	**d_{33} (pC/N)**		**T_c (°C)**
	**Thin Films**	**Bulk**	
PZT	100-150 (SI ref 4)	390-510 (SI ref 12)	300–400
**KNN**	**74-128 (SI ref 5&6)**	**300-690 (SI ref 13)**	**350**
$BaTiO_3$	100 (SI ref 7)	250-500 (SI ref 14)	130
ZnO	12.7 (SI ref 8)	9.93 (SI ref 15)	6
Doped ZnO (Transition Metal)	128 (SI ref 9)	110 (SI ref 16)	280-500 (SI ref 18)
AlN	6 (SI ref 10)	-	1150
PVDF-TrFE	28 (SI ref 11)	20-30 (SI ref 17)	110

From the comparison, it is clear that KNN has comparable piezoelectric performance to the most widely used piezoelectric material, PZT. KNN also possesses superior biocompatibility with regard to the cytotoxicity of its breakdown byproducts compared to PZT (*SI ref 19*). Furthermore, its high Curie temperature enables advanced fabrication techniques to create device architectures such as the piezoelectric micromachined ultrasound transducer (pMUT), which greatly enhances the piezoelectric properties of the device without poling or chemical modification. Indeed, piezoelectric devices with KNN have been fabricated, implanted, and evaluated for biocompatibility (*SI ref 20*).

In sum, other piezoelectric ceramics and polymers have biocompatible properties, such as $BaTiO_3$, ZnO, and PVDF, however, they either have inferior electromechanical efficiency and/or thermal processability compared to KNN. These design parameters make KNN the best material choice over PZT, especially for microfabricated implantable neurostimulation devices with the pMUT architecture.

Supplementary Note 2: Flexible SU-8 based pMUT vs rigid Si-based pMUT

SU-8 is a common material used for biocompatible implanted devices due to its low stiffness (Young's Modulus, 100x less than Si), photo-patternability, and encapsulation ability (SI refs 21, 22). These factors are important for implanted devices, as previous studies reported that, due to its flexibility, the mechanical

damage caused by SU- 8 needles in the rat's brain during its insertion is lower than that caused by rigid Si needles (SI ref 23). In chronic studies, SU-8 devices outperformed Si devices in minimizing tissue damage from mechanical mismatch (SI ref 24). The frequency-dependence of an edge-clamped circular pMUT can be modeled with $f = (\alpha / 2\pi r^2)\sqrt{(D_E/\rho h)}$ where $D_E = Eh^3/(12(1- \upsilon^2)$, where D_E is the flexural rigidity, α is the resonance mode constant, h is the diaphragm thickness, E is the effective Young's modulus, υ is the Poisson's ratio, ρ is the effective density of the diaphragm. As observed, to maintain a sub-MHz resonance frequency with Si, the diaphragm thickness must be reduced at the expense of insulation or piezo thickness; or the diaphragm radius must be increased significantly, which decreases the spatial resolution of the device. The precedent literature serves as the basis to make an informed decision of fabricating flexible pMUT using SU-8 instead of using rigid Si. We performed a COMSOL multiphysics simulation for resonance frequency and acoustic pressure of a Si pMUT and compared it with the results of our flexible pMUT as shown in supplementary fig. 5. As seen from the simulation, for the same device dimension, the SU-8-based pMUT shows a dominant resonance frequency at 545 kHz whereas Si-based pMUT has resonance at 1350 kHz. It is well established in the literature that robust stimulation in the brain occurs at a sub-MHz frequency range ideally around 500 kHz (refs 15, 22, 23, 24 in the manuscript) which helped in choosing SU-8 based neurostimulator design. In addition, as seen in Supplementary Fig 5b, the acoustic pressure exerted by both probes at their resonance frequency is almost similar, yet SU-8-based probes have the obvious advantage of mechanical flexibility as discussed above. Indeed, it is possible to reduce the resonance frequency of a pMUT structure simply by increasing the diameter of the cavity (supplementary fig. 4), however, to reduce the resonance frequency of Si-based pMUT to ~500 kHz needs significant increase in cavity size thereby overall device size (SI ref 25). Considering all this, we have decided to fabricate flexible SU-8 based pMUT instead of rigid Si-based pMUT.

Supplementary Note 3: Potential mechanisms of action of Ultrasound Neuromodulation

The ability of ultrasound waves to activate neural cells of various types has been demonstrated in several past works with explorations into mechanisms encompassing the activation of mechano-sensitive PIEZO and TRP channels, demonstrated in vitro (refs 11, 14 in manuscript) and in vivo with transcranial focused ultrasound (ref 14 in manuscript and SI ref 26). These channels exist in both neurons and astrocytes, but studies have shown that different neural cell types might respond differently to US stimulation. Zhu et al. (2023) demonstrated that knocking out the highly mechano-sensitive PIEZO channels in neurons resulted in the loss of US modulation sensitivity while knocking out PIEZO channels in astrocytes did not (SI ref 13). Lee et al. (2023) and Oh et al. (2019) demonstrated that TRPA-channels from astrocytes can be ultrasonically activated and are sufficient to indirectly excite neurons via glutamate release (Ref 14 in manuscript and SI ref 27). Therefore, we hypothesize that ultrasound can activate both neurons and

astrocytes, although likely employing different mechanisms. Indeed, the ultrasound frequency and stimulation parameters can potentially be tuned to achieve a degree of cell selectivity as has been demonstrated by other groups (ref 61 in the manuscript). Genetic or pharmacological studies that disable mechanosensitive ion channels simultaneously affect other physiological processes that maintain cell or organism viability (SI ref 28). With the development of new sonogenetic tools that enhance mechanosensitivity without disabling channel activity (SI ref 29) and that maintain a diverse environment of mechanosensitive cells (SI ref 30), the future direction of this work would include studies on how the neuronal activation is produced by the ImPULS.

In our study, the calcium indicator GCaMP7F labels primary excitatory neurons, and we demonstrated activation of excitatory cells when the transducer is placed adjacent to the neuron bodies of granule cells in the dentate gyrus (supplementary fig. 18). It is important to note, however, that its expression does not imply the lack of activation of astrocytes/glial cells. We achieved robust stimulation using an ultrasound driving protocol that is known to excite neurons (refs 23, 60 in manuscript), but the mechanism of action can be partially driven by indirect excitation via astrocytes ’gliotransmitters release.

In the scope of this work, we have demonstrated the ability of a new implantable and spatially precise device to cause neuron excitation, and we envision that further studies can help elucidate the mechanism of action of this activation in the various regions we tested, whether via direct or indirect stimulation.

Supplementary Note 4: Rationale for Ultrasound parameters

The driving frequency of 500 kHz in water is well described in the literature as an appropriate frequency for transcranial neuromodulation in mice brains (refs 15, 22, 23 and 24 in manuscript), as it presents a good tradeoff between skull transmission (more efficient in ranges below 1 MHz) and spatial selectivity (focus size decreases with frequency increase). Although our proposed implantable device bypasses the skull and is focused in a volume <100 μm^3, we chose to design a device with 500 kHz resonant frequency as it could reproduce or explore similar established protocols for neuron activation. Ye et al. (2016) investigated the frequency dependence of ultrasound neuromodulation in the mouse brain, and in the range of 0.3 - 2.9 MHz, demonstrated that the activation success rate was nearly flat at lower frequencies but, at higher frequencies, higher spatial peak intensities were necessary to attain comparable success rates when contrasted with lower ones (ref 16 in manuscript).

The Pulse Repetition Frequency dependency on ultrasound neuromodulation was explored by Manuel et al. (2020) , who demonstrated that pulsed stimulation was more efficient than continuous wave stimulation (ref 62 in the manuscript). Among pulsed US, the parameter space that led to the biggest activation of neurons (quantified by calcium imaging) had PRF of 1500 Hz, center frequency of 500 kHz, acoustic pressure of 100 kPa, and burst duty cycle of 60%, which are very similar to our stimulation parameters.

Pressures close to 100 kPa have been shown to activate neural circuitry, as exemplified by Tufail et al. (2010) (ref 22 in the manuscript), who tested pulsed driving frequencies in the 0.25-0.5 MHz range with max pressure of 97 kPa and published a separate protocols paper (ref 21 in the manuscript). Notably, they found that their pulsed protocol with PRFs in the 2.5 kHz range was sufficient to activate robust stimulation in hippocampal circuits. In other studies by Yoo et al. (2022) (ref 11 in the manuscript) in cortical neurons, pressures exceeding 150 kPa were needed to reliably drive neural activation but subthreshold pressures still had modulatory effects.

Taken together, we can see there are a number of parameter investigations for the ultrasonic modulation of neuronal tissue, but no consensus on which optimal parameters should be used. Recommendations for successful parameters of ultrasound neuromodulation encompass a range, and our chosen parameters are contained in the recommendations of both Blackmore et al.'s (2019) review and Tufail et al.'s (2011) protocol. Often the choice of parameters is limited by the availability of commercial ultrasound probes or the manufacturing capabilities of research facilities. One advantage of our chosen method of pMUT fabrication is the possibility of creating an ultrasound element or an array of elements in many sizes and center frequencies, a flexibility that will allow for the continuation of parameter investigations in various ultrasound modulation applications. Therefore, for the current work investigating neuron excitation in mice, our ultrasound driving parameters of 500 kHz, 10-50% duty cycle, 1500 Hz pulse repetition frequency are consistent with literature and corroborated our findings of ImPULS's ability to elicit a modulatory response.

Supplementary Note 5: COMSOL simulation parameters

The geometric parameters used in the COMSOL simulation model:

Layer	Material	Radius (µm)	Thickness (µm)
Passivation layer	SU-8	70	0.5
Top electrode	Au	30	0.25
Piezoelectric layer	KNN	50	1
Bottom electrode	Pt	50	0.25
Membrane layer	SU-8	70	1
Cavity	-	50	20
Backing layer	SU-8	70	20

The properties of materials used in the COMSOL simulation model:

**Properties**	**SU-8**	**Au**	**Pt**	**KNN**
Density(Kg/m^3)	1190	19300	21450	4000
Young's modulus (GPa)	4.02	70	168	65
Poisson's ratio	0.22	0.44	0.38	0.3
Piezoelectric coefficient (C/m^2)				12
Relative permittivity				1500

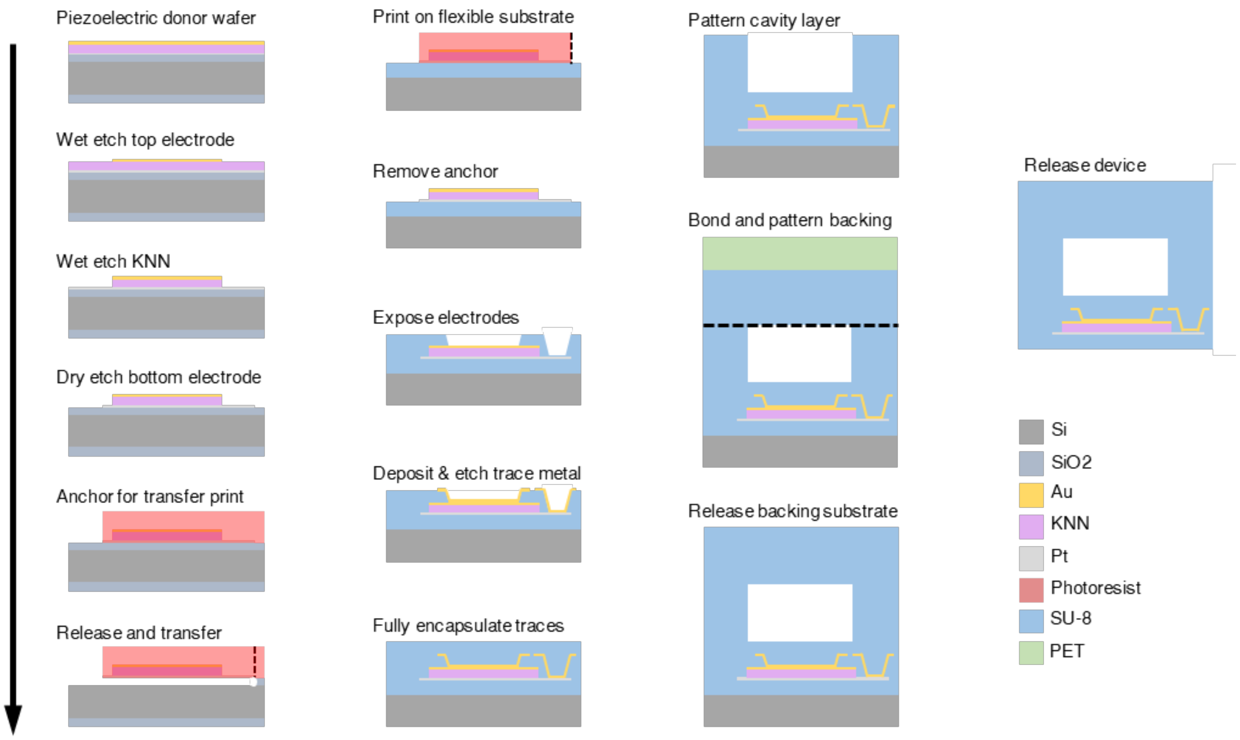

Supplementary Figure 1 | Schematic illustration of microfabrication of the ImPULS.

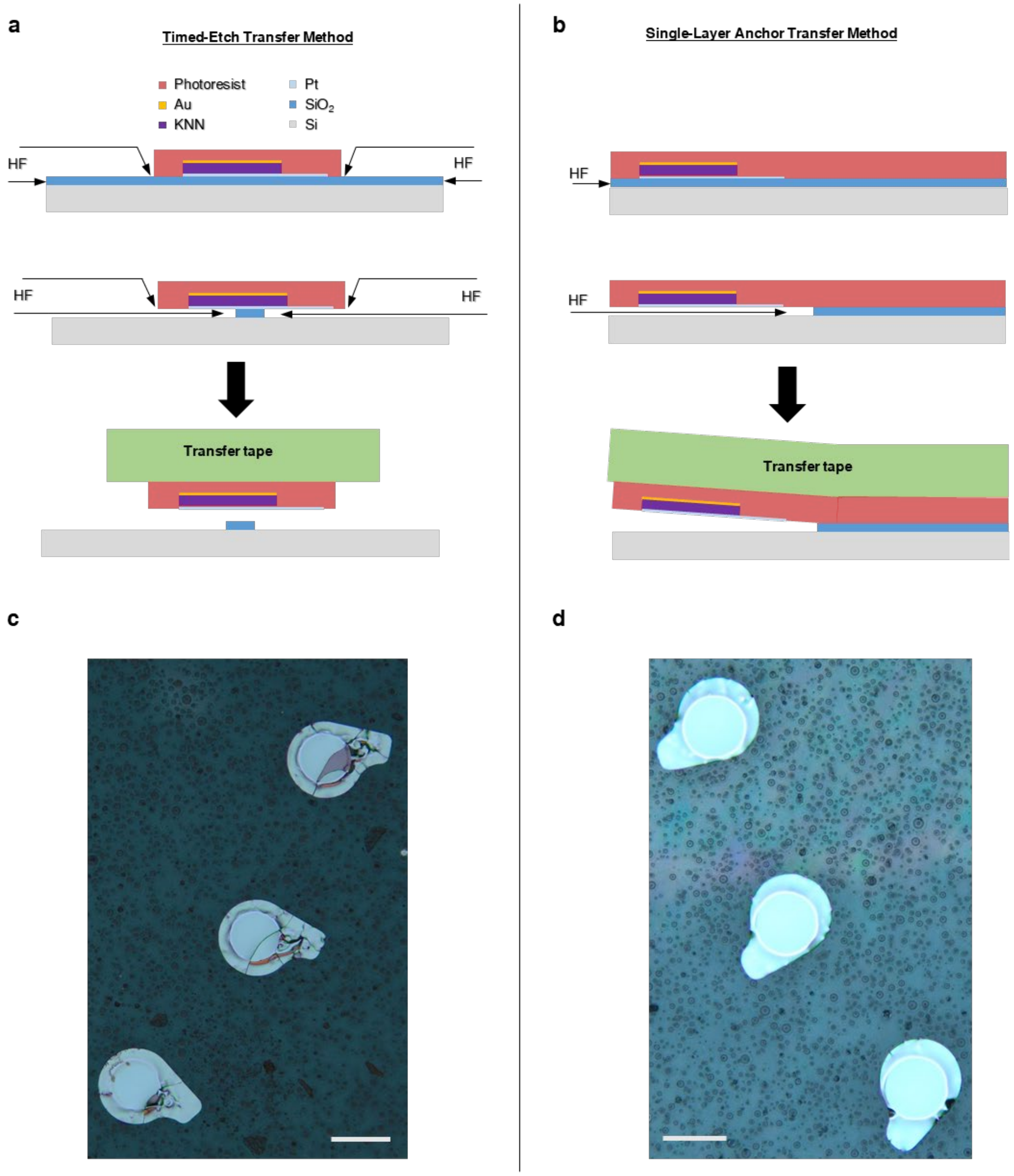

Supplementary Figure 2 | Comparison of transfer printing using two different types of anchor layer patterning. a, Timed-etch transfer method **b,** Single-layer anchor transfer method **c,** Microscopic image of time-etch transferred pattern. This type of transfer creates cracks on the bottom Pt electrode. Scale bar, 100 µm. **d,** Microscopic image of Single-layer anchor transfer pattern. No crack forms on bottom electrode during transfer. This method is less time sensitive, therefore improving the yield significantly. Scale bar, 100 µm.

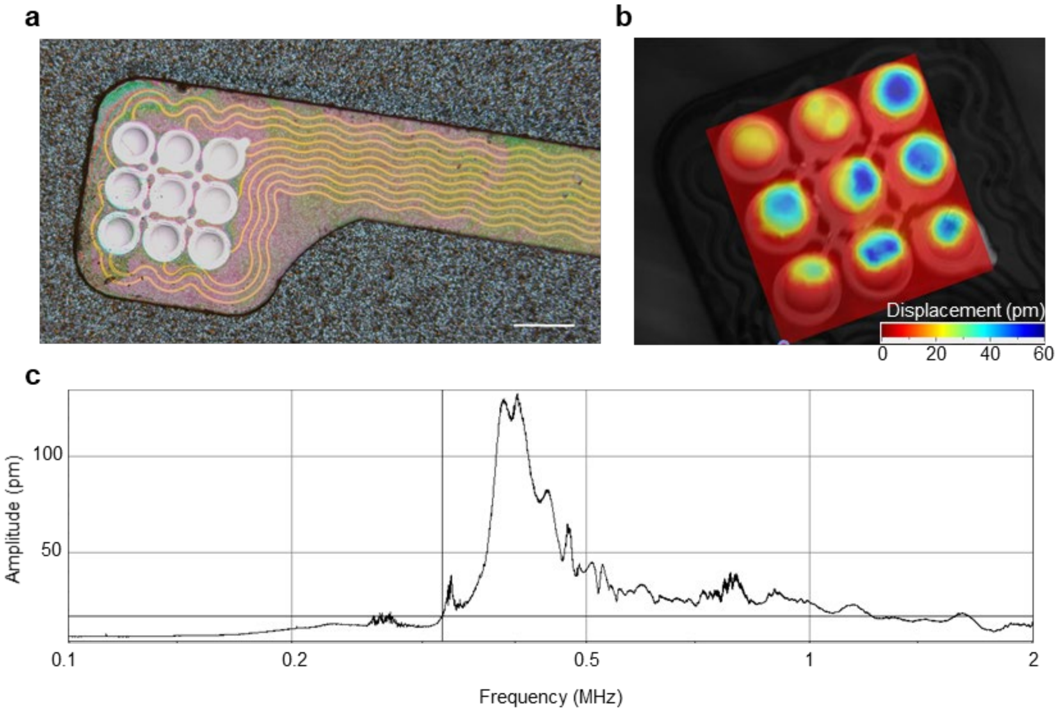

Supplementary Figure 3 | a, Microscopic image of microfabricated array with 9 piezoelectric elements. Scale, 200 μm. **b**, Laser doppler vibrometer (LDV) response of 9 elements under a periodic chirp excitation in the 0 - 2 MHz range, showing the displacement of the unique elements is in phase. **c**, Average frequency response of the array elements, showing resonant frequency close to 500 kHz.

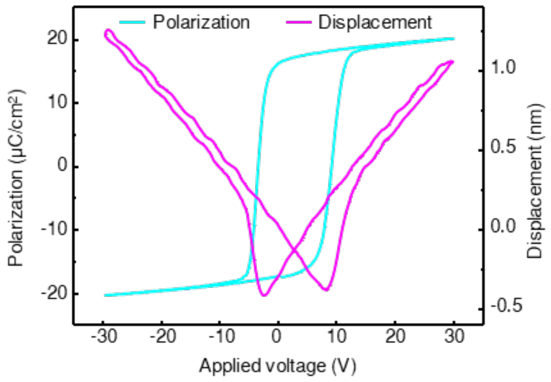

Supplementary Figure 4 | P-E hysteresis loop and piezoelectric displacement butterfly loop of KNN measured on donor wafer.

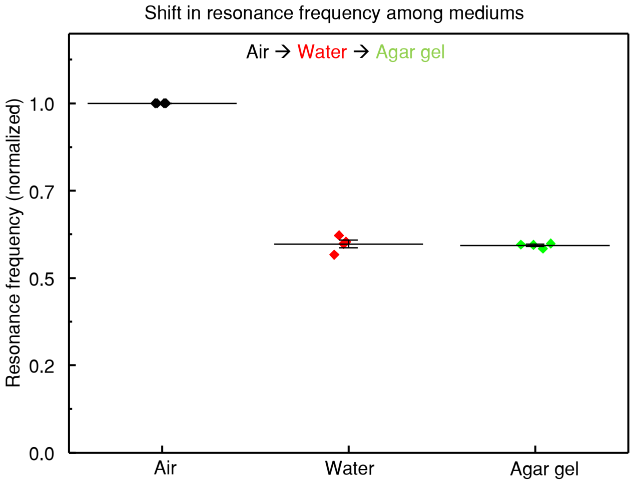

Supplementary Figure 5 | Shift in resonance frequency in different mediums (air, water and 0.6% agarose gel). Negligible shift in frequency is observed in water versus agarose gel medium. N = 4.

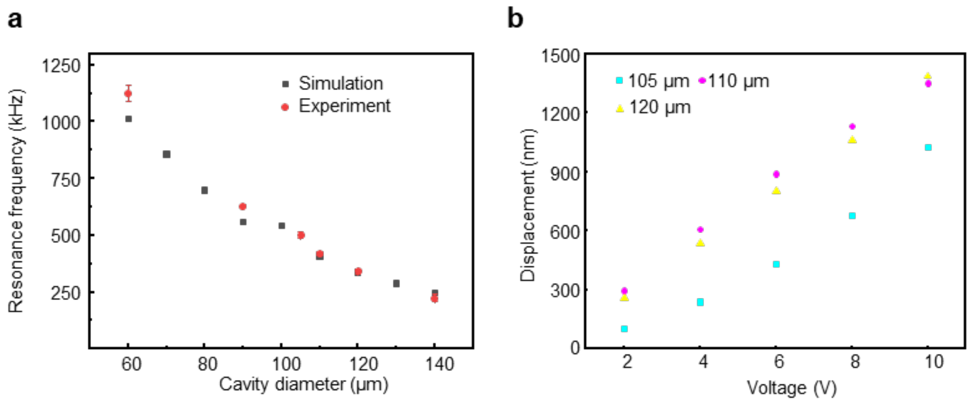

Supplementary Figure 6 | Effect of cavity size on resonance frequency and displacement of device a, Resonance frequency for varied cavity diameters comparing simulated and experimental results. **b,** Measured displacement with LDV of devices with varied cavity size and applied voltages.

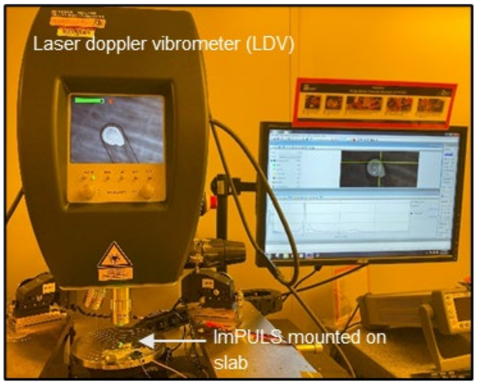

Supplementary Figure 7 | Experimental setup for displacement measurement using laser doppler vibrometer (LDV). The test device was mounted on a 3-axis stage for positioning under microscope for LDV measurement.

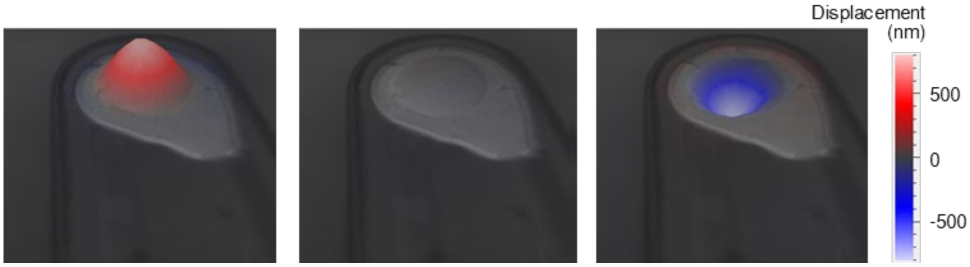

Supplementary Figure 8 | Three representative stages of membrane vibration upon application of sinusoidal signal at 10V (p-p) at fundamental resonance frequency.

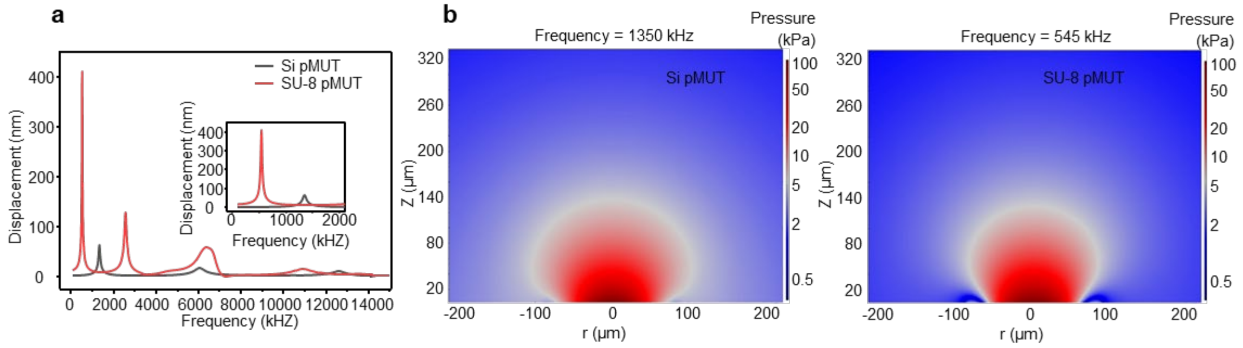

Supplementary Figure 9 | COMSOL Multiphysics simulation of Si-based and SU-8-based pMUT. a, Displacement vs frequency of Si and SU-8-based pMUT showing their resonance behavior. **b**, Simulated acoustic pressure profile showing a spherical pressure distribution.

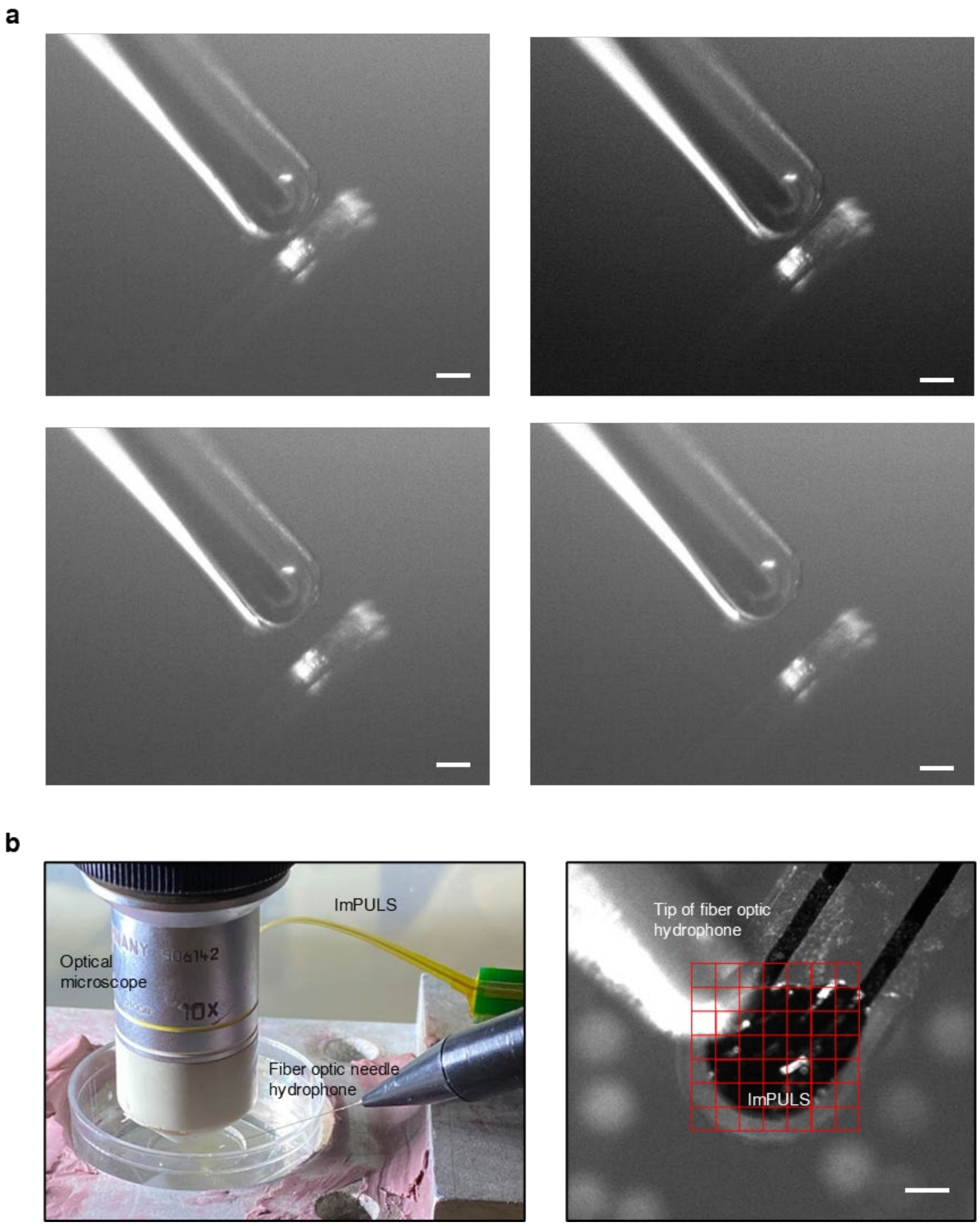

Supplementary Figure 10 | Experimental setup for ultrasound pressure measurement using needle type fiber optic hydrophone. a, Microscopic image (10x magnification) for pressure measurement showing four different distances between the probe tip and needle hydrophone. Scale bar, 50 µm. **b,** Setup for 2-D pressure and temperature mapping (left) and corresponding microscopic image (10x magnification). The red grid represents approximate measurement position of needle hydrophone. Scale bar, 50 µm.

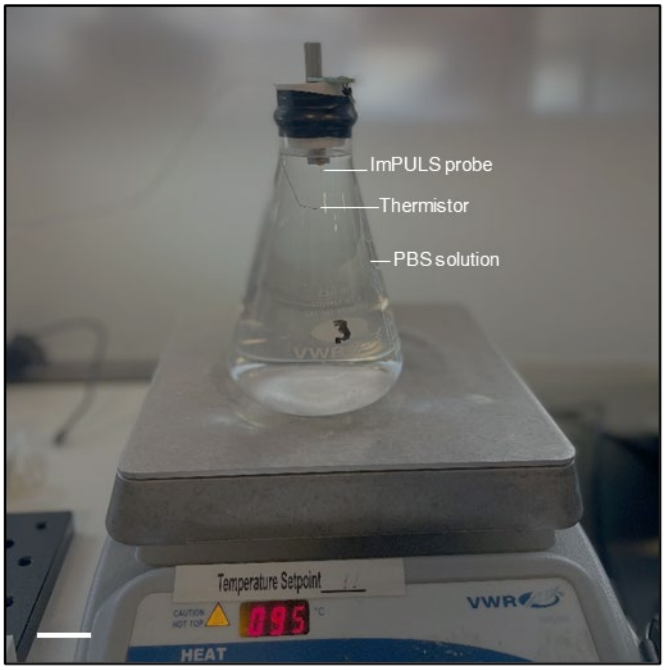

Supplementary Figure 11 | Aging test setup of ImPULS. Hotplate temperature was adjusted to 95˚C to maintain the temperature of PBS solution constant at 75˚C which was measured using a thermistor. Scale bar, 2 cm.

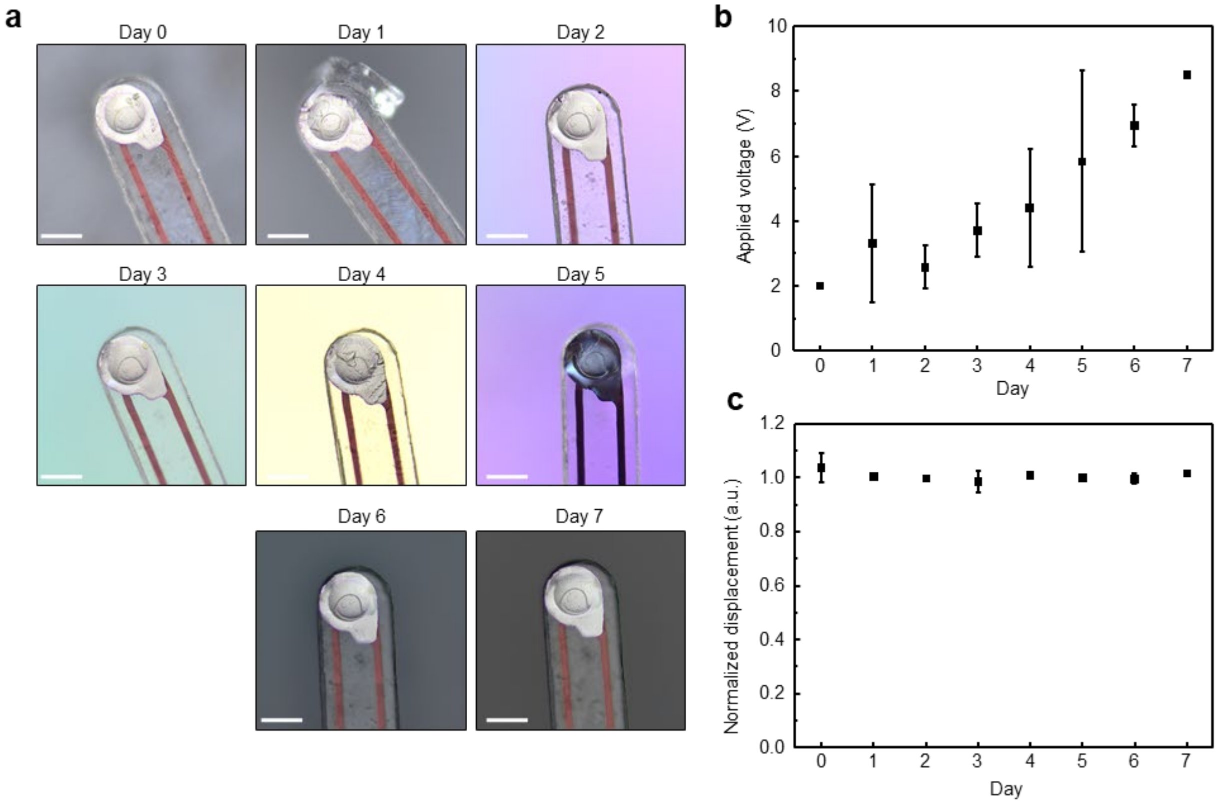

Supplementary Figure 12 | Aging test with adaptive voltage. a, Microscopic image of ImPULS taken each 24 h apart during aging test with adaptive voltage. Scale bar, 100 µm. **b**, Required applied voltage to maintain the displacement of ImPULS at 120 nm in 7 days. Error bar represents standard deviation in measurements, N = 3. **c**, Normalized displacement in 7 days. Displacement was normalized by dividing the measured displacement by 120 nm, which was the target displacement to be maintained throughout the experiment. Error bar represents standard deviation in measurements, N = 3 (a.u. = arbitrary units).

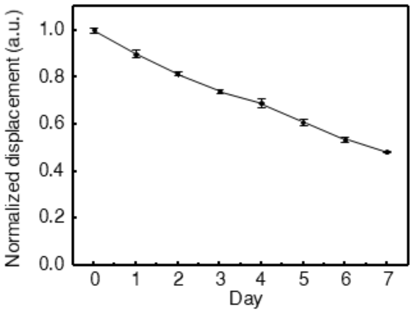

Supplementary Figure 13 | Fatigue test of ImPULS. The displacement was measured as an indicator of performance over 7 days with continuous application sinusoidal signal (500 kHz, 10 V (p-p)). The device was submerged in water for 7 days with input signal continuously ON. The measurement was performed 24 h apart for 7 days (a.u. = arbitrary units).

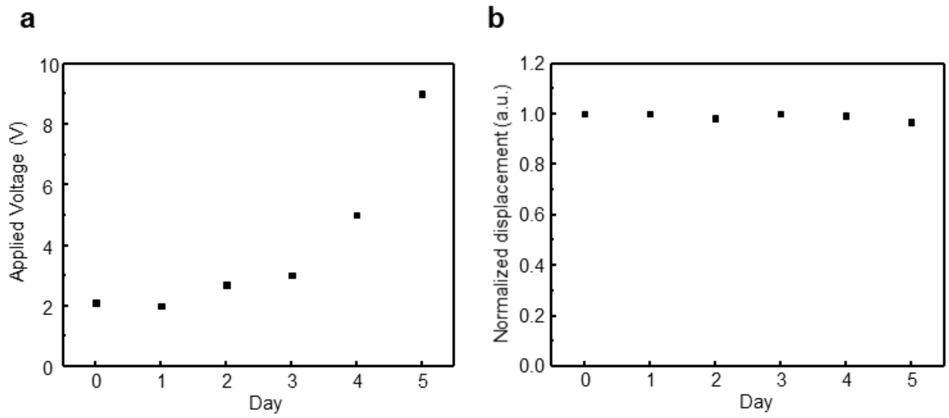

Supplementary Figure 14 | Fatigue test of ImPULS with adaptive voltage. a, Required applied voltage to maintain the displacement of ImPULS at 120 nm in 5 days. N = 1. **b**, Normalized displacement in 5 days. Displacement was normalized by dividing the measured displacement by 120 nm, which was the target displacement to be maintained throughout the experiment (a.u. = arbitrary units).

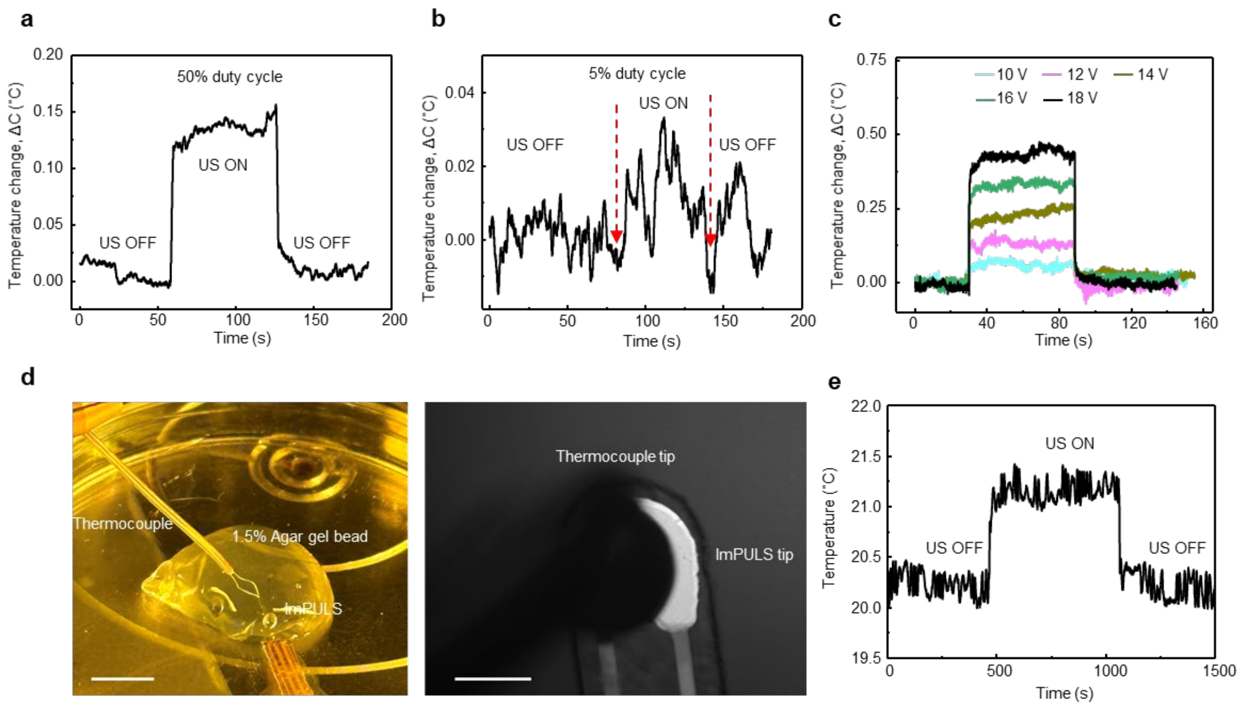

Supplementary Figure 15 | Temperature stability of ImPULS. Change in temperature when **a,** 50% duty cycle **b,** 5% duty cycle, pulsed signal at 500 kHz is applied. **c,** Change in temperature at different input voltages of continuous sinusoidal signal at 500 kHz. **d**, Image of thermal measurement setup showing the thermocouple and ImPULS device aligned within a 1.5% agar gel bead, scale bar, 5 mm (left), microscope image of the location the thermocouple tip was placed relative to the ImPULS device tip, scale bar, 100 µm. **e**, Change in temperature in 1.5% agarose gel medium when 483 kHz continuous sinusoidal signal at 20 V (p-p) is applied continuously for 10 min.

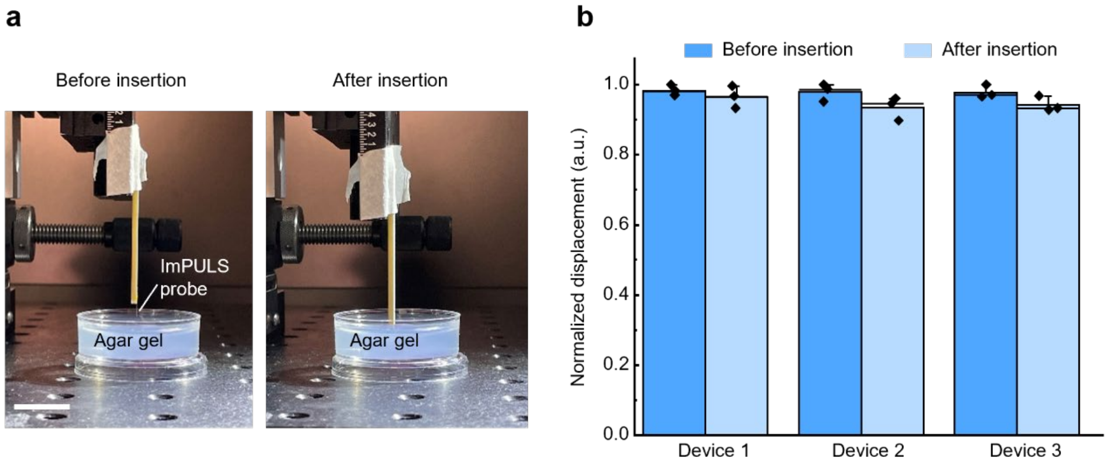

Supplementary Figure 16 | Insertion test of ImPULS to demonstrate that device performance retained after implantation. a, Optical photo of test setup for inserting ImPULS into 0.6% agarose gel. Scale bar, 2 cm. **b,** normalized displacement of ImPULS measured before and after insertion into 0.6% agarose gel (a.u. = arbitrary units). N = 3 measurement locations.

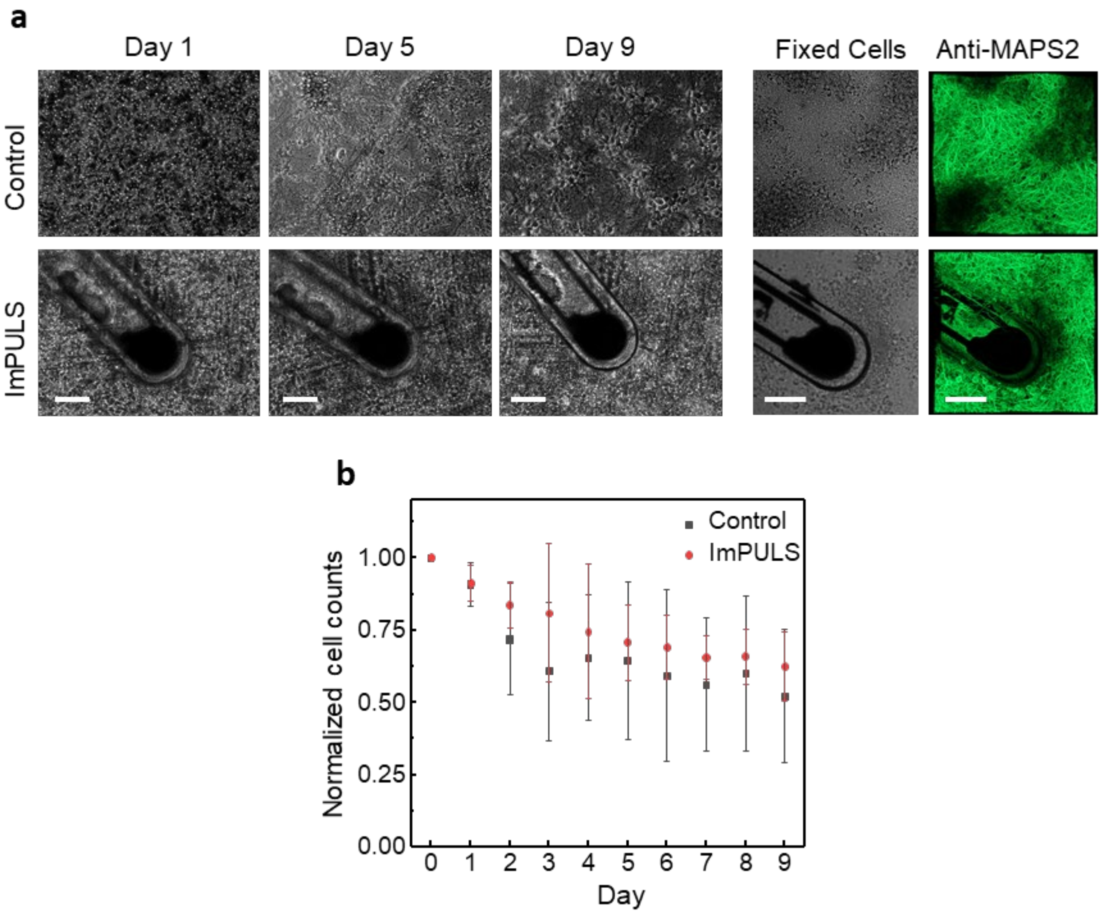

Supplementary Figure 17 | Cell Viability Study with Cultured Neurons. **a,** Representative microscopic images of cultured embryonic cortical cells on glass plates without ImPULS present and glass plates with ImPULS present over a 10 day period. After day 10, cells were fixed and stained for Anti-MAPS2 to visualize neuron somas and neurites with more contrast, Scale bar, 100 µm. **b,** Normalized cell counts over 10 days.

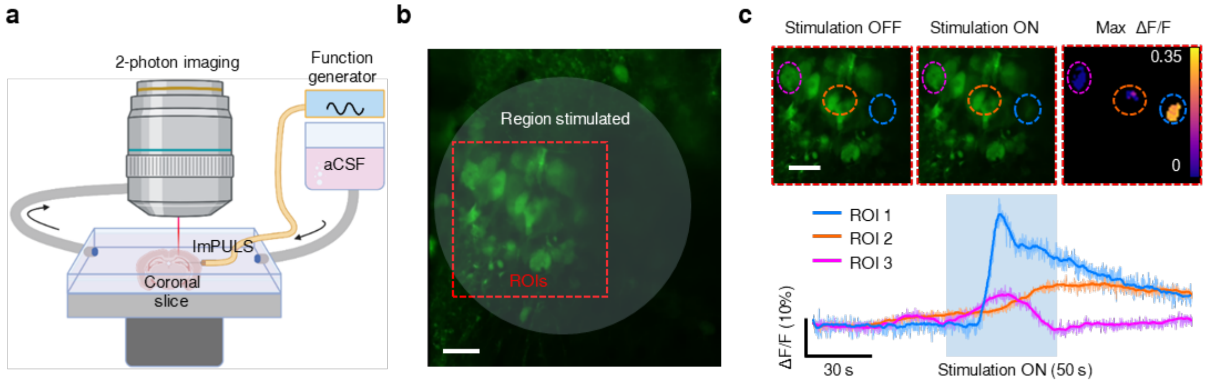

Supplementary Figure 18 | Two-photon calcium imaging of dentate gyrus in ex-vivo slice model. **a,** Schematic diagram of the two-photon imaging setup with a coronal hippocampal slice and ImPULS device under magnification. Artificial cerebrospinal fluid (aCSF) is circulated throughout the bath and a function generator connected to leads outside the bath provide the excitation for ultrasonic stimulation. Schematic created with BioRender.com, released under a Creative Commons Attribution-NonCommercial-NoDerivs 4.0 International license. **b,** View of the region of stimulation and neurons targeted (~100 µm) above the device. Scale bar, 50 µm. **c,** Averaged frames of the 'Stimulation OFF 'period preceding the 'Stimulation ON 'period showing three regions of interest (ROI). The normalized maximum fluorescence change for each ROI is depicted as an image mask and the corresponding raw traces for each ROI are shown over the course of the recording session. Scale bar, 50 µm.

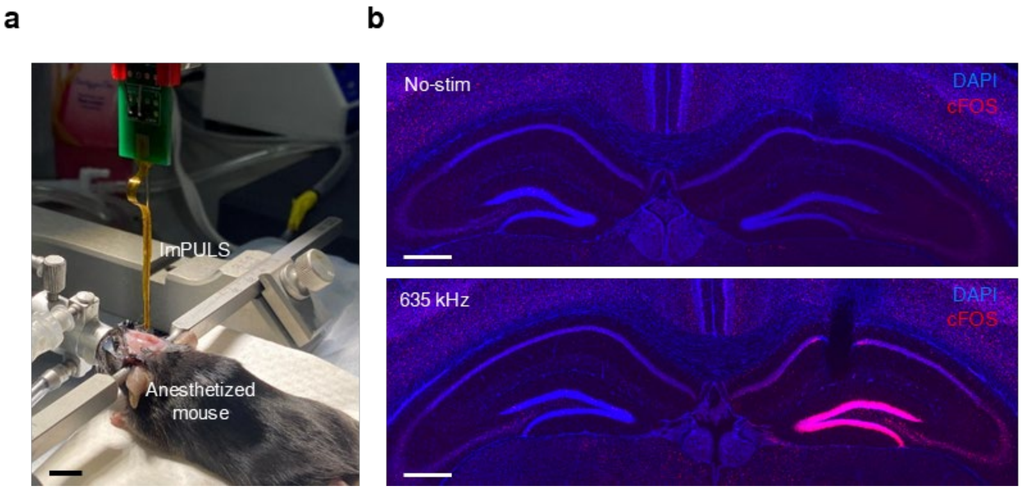

Supplementary Figure 19 | Robust stimulation of the dCA1 in anesthetized mice. a, Optical photograph of ImPULS during stereotaxic implantation procedure into the dCA1 hippocampus of an anesthetized mouse. ImPULS was implanted to right hemisphere for stimulation. Scale bar, 1 cm. **b,** Representative histology depicting a cross-section of the hippocampus during different experimental conditions: No-stim (Top), 635 kHz for 60s (Bottom). The probe tract can be visualized in the right hippocampus and DAPI+ cells are depicted in blue and cFos+ cells were labeled in red. The left hemisphere was used as negative control for immunohistochemistry. Scale bar, 500 µm.

a

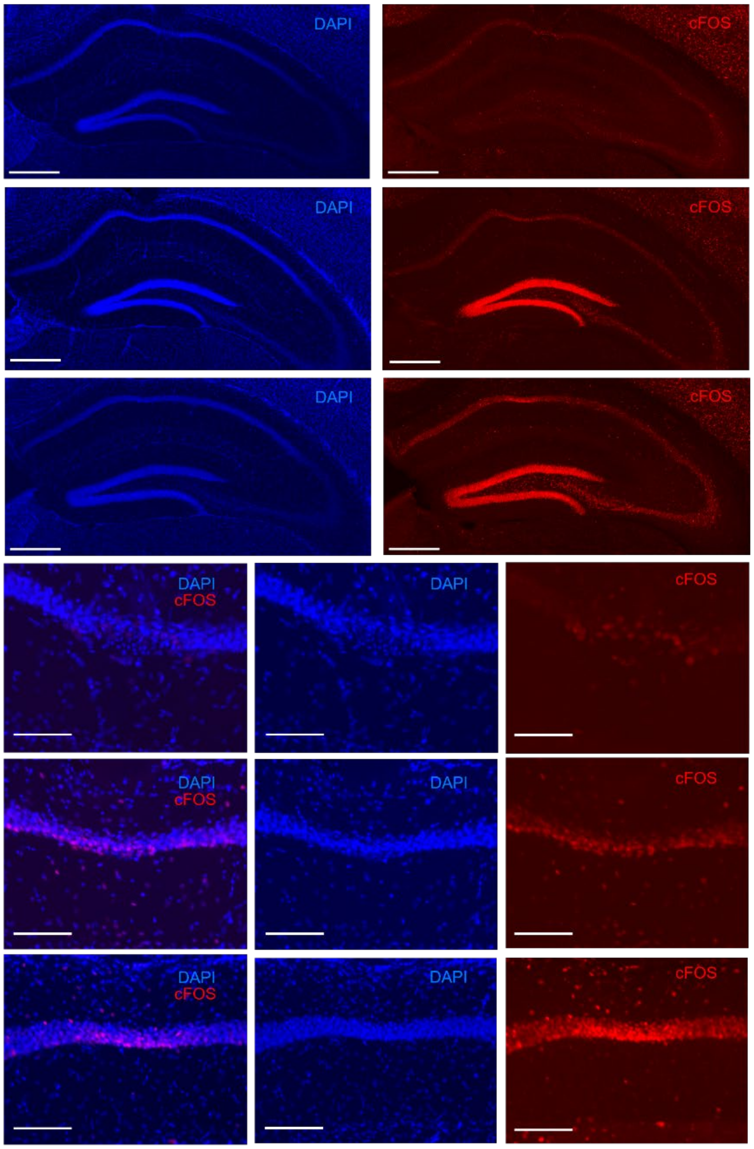

b

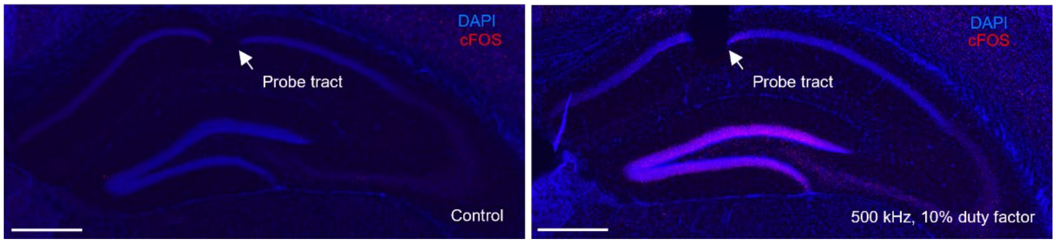

Supplementary Figure 20 | Robust stimulation of the dCA1 in anesthetized mice. a, Color-channel separated images by DAPI (left) and cFos (right) of the representative images shown in Fig. 3B. Scale bar, 450 µm. Merge (left), DAPI (middle), and cFos (right) of the representative magnified images shown in Fig. 3B. Scale bar, 100 µm. **b,** Representative histology of a tissue void depicting the probe tract of ImPULS and the termination of the stimulation location.

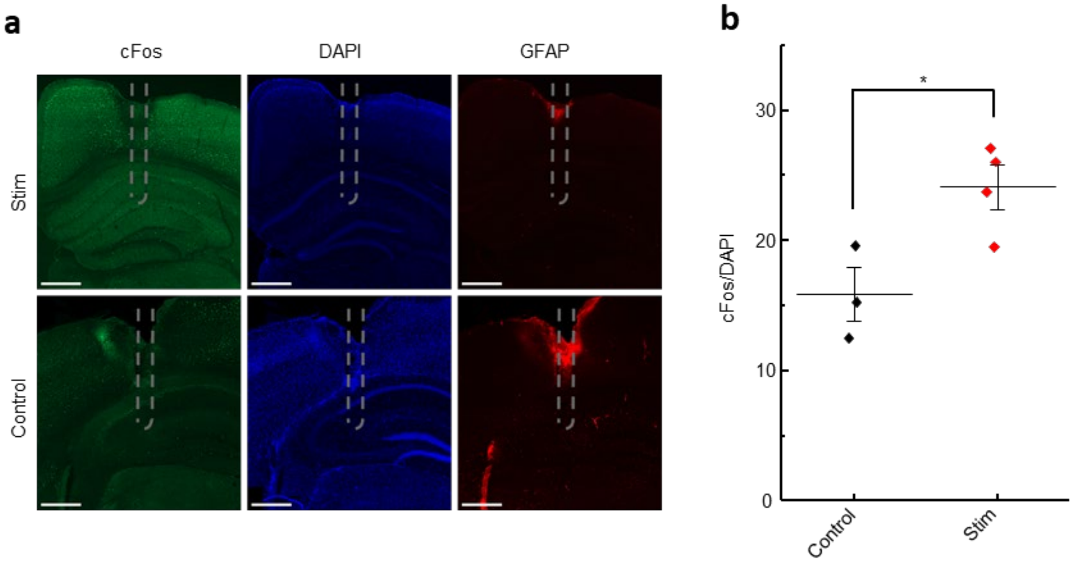

Supplementary Figure 21 | Activated cells after chronic implantation and terminal stimulation. a, Representative fluorescence images of cFos, DAPI, and GFAP in a Control Group with ImPULS implanted but no stimulation and a Stimulation Group with ImPULS implanted and a 60 s stimulation after 14 days. Stimulation occurs at the end of the estimated device profile shown in dotted lines, Scale bar, 500 µm. **b**, Cell counts for cFos+ cells normalized to DAPI+ cells in dCA1 following stimulation after a period of 14 days post-implantation (N = 3-4 mice per condition; No-stim vs. 500 kHz, 10% duty factor: unpaired t-test p = 0.0258).

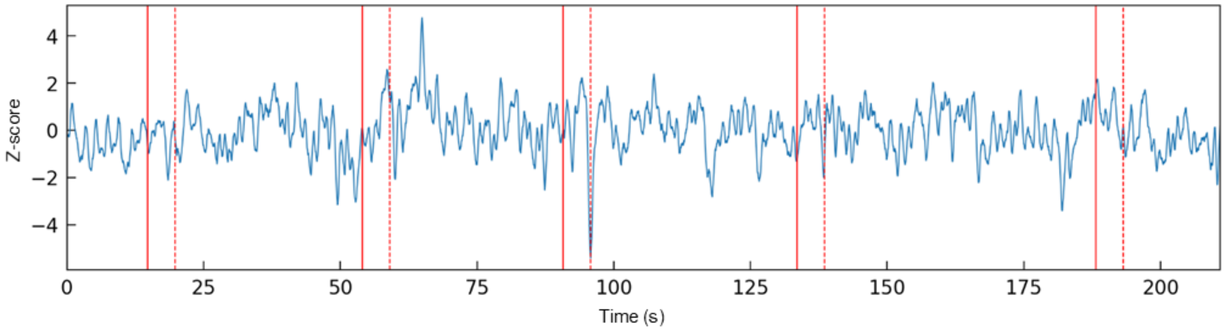

Supplementary Figure 22 | Waveform of stimulation in location approximately 200 μm dorsal to the target stimulation location (control).

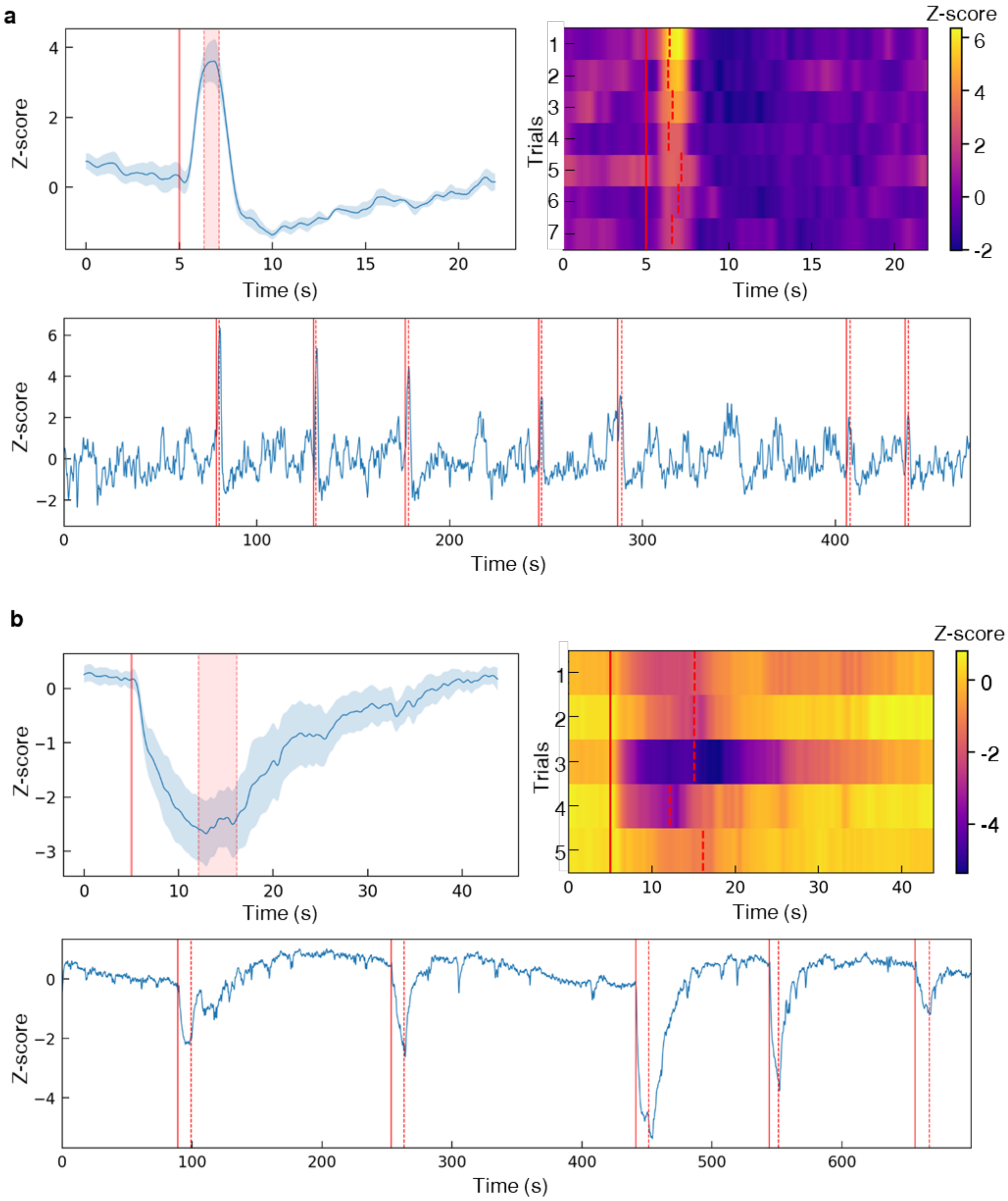

Supplementary Figure 23 | Excitatory and inhibitory modulation of nigrostriatal dopamine release in anesthetized mice. a, For mouse 4, averaged DA2m fluorescence responses for SNc and heatmap showing relative Z-score of each stimulation trial (top). First solid red line indicates stimulation start time. Shaded region bounded by dotted lines indicates range of stimulation end times. Full recording trace of Z-score DA2m fluorescence during stimulation trial (bottom) **b,** For mouse 5, averaged DA2m fluorescence responses for SNc and heatmap showing relative Z-score of each stimulation trial (top). First solid red line indicates stimulation start time. Shaded region bounded by dotted lines indicates range of stimulation end times. Full recording trace of Z-score DA2m fluorescence during stimulation trial (bottom).

Supplementary References:

1. Tokay O. , Yazıcı M. A review of potassium sodium niobate and bismuth sodium titanate based lead free piezoceramics. *Materials Today Communications*. **31**, 103358, (2022).

2. Yang J., et al. Large piezoelectric properties in KNN-based lead-free single crystals grown by a seed-free solid-state crystal growth method. *Applied Physics Letters* **108.18**, 182904, (2016).

3. Hu C., et al. Ultra-large electric field–induced strain in potassium sodium niobate crystals. *Science advances* **6.13**, eaay5979 (2020).

4. Herdier, R., Jenkins, D., Remiens, D., Dupont, M. and Osmont, D. A silicon cantilever beam structure for the evaluation of d31, d33 and e31 piezoelectric coefficients of PZT thin films. *Sixteenth IEEE International Symposium on the Applications of Ferroelectrics, Nara, Japan*, 725-727 (2007).

5. Zang, G.-Z., Yi, Z.-J., Du, J., Wang, Y.-F. Co_2O_3 doped $(Na_{0.65}K_{0.35})NbO_3$ piezoceramics. *Materials Letters.* **64(12)**, 1394-1397 (2010).

6. Egerton, L. and Dillon, D.M. Piezoelectric and Dielectric Properties of Ceramics in the System Potassium—Sodium Niobate. *Journal of the American Ceramic Society*. **42**, 438-442 (1959).

7. Acosta, M. et al. BaTiO3-based piezoelectrics: Fundamentals, current status, and perspectives. *Appl. Phys. Rev.* **4 (4)**, 041305 (2017).

8. Li, Y. et al. Towards high-performance linear piezoelectrics: Enhancing the piezoelectric response of zinc oxide thin films through epitaxial growth on flexible substrates. *Applied Surface Science.* **556,** 149798 (2021).

9. Pan, F., Song, C., Liu, X.J., Yang, Y.C., Zeng, F. Ferromagnetism and possible application in spintronics of transition-metal-doped ZnO films. *Materials Science and Engineering: R: Reports.* **62 (1)**, 1-35 (2008)

10. Anggraini, S.A., Uehara, M., Hirata, K., Yamada, H. and Akiyama, M. Polarity Inversion of Aluminum Nitride Thin Films by using Si and MgSi Dopants. *Sci Rep.* **10**, 4369 (2020).
11. Hu, X., You, M., Yi, N., Zhang X. and Xiang Y. Enhanced Piezoelectric Coefficient of PVDF-TrFE Films via In Situ Polarization. *Front. Energy Res.* **9** (2021).
12. Kim, S., and Lee, H. Piezoelectric Ceramics with High d33 Constants and Their Application to Film Speakers. *Materials (Basel, Switzerland).* **14(19)**, 5795 (2021).
13. Zhang, N., Zheng, T. and Wu, J. Lead-Free (K,Na)NbO3-Based Materials: Preparation Techniques and Piezoelectricity. *ACS Omega*. **5 (7)**, 3099-3107 (2020).
14. Dai, B. et al. Piezoelectric grain-size effects of BaTiO3 ceramics under different sintering atmospheres. *J Mater Sci: Mater Electron.* **28**, 7928–7934 (2017).
15. Zhao, M.-H., Wang, Z.-L., & Mao, S. X. Piezoelectric characterization of individual zinc oxide nanobelt probed by piezoresponse force microscope. *Nano Letters*, ***4*(4)**, 587–590 (2004).
16. Yang, Y. C., Song, C., Wang, X. H., Zeng, F., Pan, F. Giant piezoelectric d_{33} coefficient in ferroelectric vanadium doped ZnO films. *Appl. Phys. Lett.* **92 (1)**, 012907 (2008).
17. Zhang, L. et al. Recent Progress on Structure Manipulation of Poly(vinylidene fluoride)-Based Ferroelectric Polymers for Enhanced Piezoelectricity and Applications. *Adv. Funct. Mater.* **33**, 2301302 (2023).
18. Straumal, B. B. et al. Ferromagnetic behaviour of ZnO: the role of grain boundaries. *Beilstein journal of nanotechnology.* **7** 1936-1947 (2016).
19. Jeong C. K., et al. Comprehensive biocompatibility of nontoxic and high-output flexible energy harvester using lead-free piezoceramic thin film. *APL Mater*. **5**, 074102 (2017).
20. Chen W., et al. Fabrication of Biocompatible Potassium Sodium Niobate Piezoelectric Ceramic as an Electroactive Implant. *Materials (Basel).* **10(4)**, 345 (2017).
21. Nemani K. V., Moodie K. L. , Brennick J. B., Su A., Gimi B. In vitro and in vivo evaluation of SU-8 biocompatibility. *Mater Sci Eng C Mater Biol Appl*. **33(7)**, 4453-9, (2013).

22. Zhao S., et al. Tracking neural activity from the same cells during the entire adult life of mice. *Nat Neurosci.* **26**, 696–710, (2023).

23. Fernández L. J., et al. Study of functional viability of SU-8-based microneedles for neural applications. *J. Micromech. Microeng.* **19(2)**, 025007, (2009).

24. Huang S-H., Lin S-P. , Chen J-J. J. In vitro and in vivo characterization of SU-8 flexible neuroprobe: From mechanical properties to electrophysiological recording. *Sensors and Actuators A: Physical.* **216**, 257-265, (2014).

25. Qiu Y., et al. Piezoelectric Micromachined Ultrasound Transducer (PMUT) Arrays for Integrated Sensing, Actuation and Imaging. *Sensors*. **15(4)**, 8020-8041, (2015).

26. Zhu J., et al. The mechanosensitive ion channel Piezo1 contributes to ultrasound neuromodulation. Proc Natl Acad Sci USA. 120(18), e2300291120, (2023).

27. Lee K., et al. Ultrasonocoverslip: In-vitro platform for high-throughput assay of cell type-specific neuromodulation with ultra-low-intensity ultrasound stimulation. Brain Stimulation. 16(5), 1533-1548, (2023).

28. Dubin, A.E., Schmidt, M., Mathur, J., Petrus, M.J., Xiao, B., Coste, B., and Patapoutian, A. Inflammatory signals enhance piezo2-mediated mechanosensitive currents. Cell Rep. 2, 511–517 (2012).

29. Cadoni, S. et al. Ectopic expression of a mechanosensitive channel confers spatiotemporal resolution to ultrasound stimulations of neurons for visual restoration. Nat. Nanotechnol. 18, 667–676 (2023).

30. Newman, M. et al. Ultrasound Modulates Calcium Activity in Cultured Neurons, Glial Cells, Endothelial Cells and Pericytes. Ultrasound in Medicine & Biology. 50(3), 341-351 (2024).
