# Supplementary material for: An implantable piezoelectric ultrasound stimulator (ImPULS) for deep brain activation
Source: Nat Commun. 2024 Jun 4;15:4601. doi: 10.1038/s41467-024-48748-6 (PMC11150473; doi:10.1038/s41467-024-48748-6)
Supplement: Supplementary file 3 — Description of Additional Supplementary Files [file 41467_2024_48748_MOESM3_ESM.pdf]

Description of Additional Supplementary Files

Supplementary Movie 1 - Video (4x) of Calcium imaging session recorded by two-photon microscopy. Red arrow showing ROI 1. Original video was recorded at frame of 8.02.
