# Supplementary material for: An implantable piezoelectric ultrasound stimulator (ImPULS) for deep brain activation
Source: Nat Commun. 2024 Jun 4;15:4601. doi: 10.1038/s41467-024-48748-6 (PMC11150473; doi:10.1038/s41467-024-48748-6)
Supplement: Supplementary file 5 — Reporting Summary [file 41467_2024_48748_MOESM5_ESM.pdf]

nature portfolio

Corresponding author(s): Canan Dagdeviren

Last updated by author(s): Apr 30, 2024

Reporting Summary

Nature Portfolio wishes to improve the reproducibility of the work that we publish. This form provides structure for consistency and transparency in reporting. For further information on Nature Portfolio policies, see our Editorial Policies and the Editorial Policy Checklist.

Statistics

For all statistical analyses, confirm that the following items are present in the figure legend, table legend, main text, or Methods section.

n/a	Confirmed	
☐	☒	The exact sample size (*n*) for each experimental group/condition, given as a discrete number and unit of measurement
☐	☒	A statement on whether measurements were taken from distinct samples or whether the same sample was measured repeatedly
☐	☒	The statistical test(s) used AND whether they are one- or two-sided<br>*Only common tests should be described solely by name; describe more complex techniques in the Methods section.*
☒	☐	A description of all covariates tested
☐	☒	A description of any assumptions or corrections, such as tests of normality and adjustment for multiple comparisons
☐	☒	A full description of the statistical parameters including central tendency (e.g. means) or other basic estimates (e.g. regression coefficient) AND variation (e.g. standard deviation) or associated estimates of uncertainty (e.g. confidence intervals)
☐	☒	For null hypothesis testing, the test statistic (e.g. *F*, *t*, *r*) with confidence intervals, effect sizes, degrees of freedom and *P* value noted<br>*Give P values as exact values whenever suitable.*
☒	☐	For Bayesian analysis, information on the choice of priors and Markov chain Monte Carlo settings
☒	☐	For hierarchical and complex designs, identification of the appropriate level for tests and full reporting of outcomes
☒	☐	Estimates of effect sizes (e.g. Cohen's *d*, Pearson's *r*), indicating how they were calculated

Our web collection on statistics for biologists contains articles on many of the points above.

Software and code

Policy information about availability of computer code

Data collection	The equipment used (described in Methods section) has native proprietary software used to collect data for these investigations.
Data analysis	Generalized data analysis and plotting was performed with Python (3.9), OriginPro, and Matlab 2023. For cell imaging and analysis ImageJ, Python (Caiman 1.10.2) and QuPath v0.5.0 were used.

For manuscripts utilizing custom algorithms or software that are central to the research but not yet described in published literature, software must be made available to editors and reviewers. We strongly encourage code deposition in a community repository (e.g. GitHub). See the Nature Portfolio guidelines for submitting code & software for further information.

Data

Policy information about availability of data

All manuscripts must include a data availability statement. This statement should provide the following information, where applicable:

- Accession codes, unique identifiers, or web links for publicly available datasets
- A description of any restrictions on data availability
- For clinical datasets or third party data, please ensure that the statement adheres to our policy

All data supporting the findings of this study are either included within the paper or available from the corresponding author upon request. Source data is provided at 10.6084/m9.figshare.25571583

Research involving human participants, their data, or biological material

Policy information about studies with human participants or human data. See also policy information about sex, gender (identity/presentation), and sexual orientation and race, ethnicity and racism.

Reporting on sex and gender	N/A
Reporting on race, ethnicity, or other socially relevant groupings	N/A
Population characteristics	N/A
Recruitment	N/A
Ethics oversight	N/A

Note that full information on the approval of the study protocol must also be provided in the manuscript.

Field-specific reporting

Please select the one below that is the best fit for your research. If you are not sure, read the appropriate sections before making your selection.

- [x] Life sciences
- [ ] Behavioural & social sciences
- [ ] Ecological, evolutionary & environmental sciences

For a reference copy of the document with all sections, see nature.com/documents/nr-reporting-summary-flat.pdf

Life sciences study design

All studies must disclose on these points even when the disclosure is negative.

Sample size	Sample sizes of 3-4 for each experimental group were used in this preliminary study to assess the effects of our novel stimulus. Due to the high effect size of the stimulation, we were able to demonstrate the effects of ImPULS within the number of subjects used.
Data exclusions	No data were excluded from analysis.
Replication	1 mouse from the cohort that received stimulation in our DA photometry experiments showed an inhibitory DA signal with the application of stimulation from ImPULS. Sufficient explanation of this result can be found in the 'Discussion' section of the main text.
Randomization	Mice in experimental cohorts for cFos experiments were chosen at random to receive either 'Control' (No stimulation) or positive stimulation from the ImPULS device. Randomization in the photometry study is not relevant due to our use of an internal control.
Blinding	Blinding was not relevant to this study due to the fact that stimulation from ImPULS was either applied (Stim condition) or not applied (Control). Investigators were not blinded to the groups during experiments.

Reporting for specific materials, systems and methods

We require information from authors about some types of materials, experimental systems and methods used in many studies. Here, indicate whether each material, system or method listed is relevant to your study. If you are not sure if a list item applies to your research, read the appropriate section before selecting a response.

Materials & experimental systems

n/a	Involved in the study	
	x	Antibodies
x		Eukaryotic cell lines
x		Palaeontology and archaeology
	x	Animals and other organisms
x		Clinical data
x		Dual use research of concern
x		Plants

Methods

n/a	Involved in the study	
x		ChIP-seq
x		Flow cytometry
x		MRI-based neuroimaging

Antibodies

Antibodies used	1:500 anti-MAPS2 (Abcam), 1:500 Alexa Fluor 488 (Abcam), 1:1,000 rabbit anti-cFos (Synaptic Systems), 1:1000 chicken anti-GFP (Invitrogen), 1:200 Alexa 555 anti-rabbit (Invitrogen); 1:200 Alexa 488 anti-chicken (Invitrogen), NeuroTrace Nissl 435/455 (1:500, ThermoFisher, Cat. #N21479), rabbit anti-TH (1:1500, Millipore, Cat. #AB152), donkey anti-rabbit Alexa Fluor 647 (1:500,

	ThermoFisher, Cat. #A-31573),
Validation	All antibodies were validated by their manufacturers.

Animals and other research organisms

Policy information about studies involving animals; ARRIVE guidelines recommended for reporting animal research, and Sex and Gender in Research

Laboratory animals	Adult male and female C57BL/6J mice (The Jackson Laboratory). Embryonic day 17 C57BL/6J mice (The Jackson Labs) for cell culture.
Wild animals	No wild animals were used for these experiments.
Reporting on sex	The sex of mice is reported but not relevant to the experimental conditions or setup.
Field-collected samples	No samples were field-collected
Ethics oversight	All animal handling and experiment procedures were approved by The MIT Committee for Animal Care, the Institutional Animal Care and User Committee of the California Institute of Technology, and Boston University Institutional Animal Care and Use Committee.

Note that full information on the approval of the study protocol must also be provided in the manuscript.

Plants

Seed stocks	N/A
Novel plant genotypes	N/A
Authentication	N/A
